# The virtual interview format for fellowship recruitment in obstetrics and gynecology: a nationwide survey of program directors

**DOI:** 10.1080/10872981.2022.2054304

**Published:** 2022-03-22

**Authors:** Jane M. Ponterio, Melissa Keslar, Nisha A. Lakhi

**Affiliations:** a Office of Student Affairs, New York Medical College, Valhalla, NY, USA; b Department of Obstetrics and Gynecology, Richmond University Medical Center, Staten Island, NY, USA; c Department of Obstetrics and Gynecology, New York Medical College, Valhalla, NY, USA

**Keywords:** COVID-19, obstetrics and gynecology, subspecialty fellowship, virtual interviews, program directors

## Abstract

Due to Covid-19, fellowship programs could not conduct in-person interviews during the 2020–2021 interview cycle and were forced to implement virtual interviews. We conducted two nationwide surveys of residency and fellowship Program Directors (PDs) involved in the Obstetrics and Gynecology (Ob/Gyn) Subspecialty Fellowship match cycle to gain a better understanding of virtual interviews from each of their perspectives. 1) Fellowship PDs’ confidence in using a virtual platform to holistically evaluate applicants during the 2020–2021 match cycle, 2) Residency PD’s perception of virtual interviews and impact on their program’s operations, and 3) to assess the desire of fellowship and residency PDs to continue virtual recruitment during forthcoming interview seasons. Two separate nationwide web-based surveys were administered to 1) Ob/Gyn fellowship PDs and 2) residency PDs through SurveyMonkey from July-September 2020 to assess the impact of virtual interviews form each parties’ perspective. Surveys solicited demographic information, four-point Likert scale questions, and free response questions Of programs meeting inclusion criteria, 75/111 (67.6%) fellowship PDs and 67/117 (57.3%) residency PDs responded to their respective surveys. Most fellowship PDs believed that they could confidently assess applicants’ professionalism (88%) during a virtual interview and (90.7%) felt confident in making a rank-order list. However, only 73.3% were just as confident in preparing a rank list after a virtual interview as they have been with in-person interviews. Most residency PDs (69.9%) believed that virtual interviews made it easier for their program to comply with duty hours, and 76.8% agreed that virtual interviews allowed their residents to accept more interviews than an in-person format. Most fellowship PDs found virtual interviews convenient. However, difficulty in observing social interaction and gauging applicant interest may be the biggest challenge moving forward.

## Introduction

Obstetrics and gynecology residents applying for fellowship positions traditionally participate in face-to-face interviews with faculty members at each prospective program. This process is structured to allow applicants an opportunity to visit program sites, interact with program faculty, and socialize with the current fellows. Likewise, program directors and faculty use the interview process to assess an applicant’s academic motivations, professionalism, interpersonal skills, and overall fit for the program. However, there are several disadvantages to the traditional in-person interview format that impact residency programs, applicants, and fellowship programs. Due to required travel, in-person interviews are cumbersome to residency programs because of resident absenteeism, work-hour restrictions, disrupted program workflow, and loss of continuity of care [1]. Residency PDs often have to adjust schedules to accommodate for absenteeism during the interview season, with other residents who may not be interviewing, taking on extra workloads. For applicants, time away from residency training diminishes educational opportunity, surgical case volume, and patient care interactions [2]. Additionally, traveling to multiple out-of-town interviews may result in financial hardship for applicants [1–3].

The Covid-19 pandemic has had widespread impact upon the healthcare system, including academic medicine. The resultant travel restrictions and social distancing policies precluded applicant travel for fellowship interviews during the 2020–2021 match cycle. Interestingly, virtual conferencing interview platforms have been reported as early as 2000 for internal medicine residency interviews [4]. Since then, virtual interviews have been utilized successfully in several other settings including urology residency selection, ophthalmology, plastic and reconstructive surgery, and gastroenterology fellowship programs [5–10].

Although virtual interviews have not previously been utilized by any of the Obstetrics and Gynecology subspecialty fellowship programs, the 2020–2021 interview season presented an opportunity to evaluate its utility. We aimed to assess fellowship program directors’ perspectives surrounding the 2020–21 match season conducted virtually due to the Covid-19 pandemic. Many speculated that the virtual match process might lead to an overwhelming number of unmatched applicants and programs. However, the NRMP statistics for the 2020–2021 Ob/Gyn Fellowship match outcomes showed that the number of applicants per position, the percentage of matched applicants, and the percentage of filled programs were comparable to the previous three years [11]. Yet several issues should be carefully evaluated if virtual interviews are to become part of the ‘new normal’ for fellowship recruitment. The change of format not only directly impacted fellowship PDs and applicants, but also the applicants’ respective residency PDs and resident colleagues [12–14]. Thus, we conducted two nationwide surveys targeting both residency and fellowship Program Directors (PDs) involved in the Ob/Gyn Subspecialty Fellowship match cycle to gain a better understanding of each of their perspectives. The survey of the Fellowship PDs’ sought to evaluate their perspective of the virtual interview process as well as their confidence in choosing applicants virtually. The Residency PD survey was designed to evaluate their perception of virtual interviews and its impact on their program’s operations, including supervision, clinical coverage, and work hours compared to an in-person format. We hope that their combined perspectives may help to better shape future interview cycles.

## Materials and methods

This prospective study was conducted at New York Medical College School of Medicine (Valhalla, New York) and Richmond University Medical Center (Staten Island, New York). The study protocol (protocol number: 14,339) was reviewed by the Institutional Review Board of New York Medical College and received an IRB exemption. We conducted a nationwide survey of residency and fellowship Program Directors (PDs) accredited by the Accreditation Council for Graduate Medical Education (ACGME) involved in the Ob/Gyn Subspecialty Fellowship 2020–2021 match cycle. The objectives of our study were to evaluate 1) Fellowship PDs’ confidence in using a virtual platform to holistically evaluate applicants during the 2020–2021 match cycle, 2) Residency PD’s perception of virtual interviews and impact on their program’s operations, and 3) assess the desire of fellowship and residency PDs to continue virtual recruitment during forthcoming interview seasons.

This study was conducted by email questionnaires administered through the survey tool, Survey Monkey^TM^. Surveys were administered from July 2020 – September 2020 to Ob/Gyn subspeciality Fellowship Program Directors and Ob/Gyn Residency Program Directors. For the 2020–2021 match cycle, the Match opened on 24 June 2020, the Rank Order List Certification deadline was September 30th, 2020 and the Match results were announced on October 14th, 2020.

The Fellowship Program Directors surveyed were those from the Female Pelvic Medicine and Reconstructive Surgery (FPMRS), Gynecologic Oncology (Gyn/Onc), Maternal Fetal Medicine (MFM), and Reproductive Endocrinology and Infertility (REI) subspecialties. Email addresses of Fellowship Program Directors and Ob/Gyn residency Program Directors were gathered from the national Ob/Gyn residency program list developed by Accreditation Council for Graduate Medical Education (ACGME) using the Institutional and Program Finder tool (https://apps.acgme.org/ads/Public/Programs/Search). The generated list for each specialty was reviewed and programs that provided an email address of the Program Director were included. Exclusion criteria were programs providing email addresses for only the Program Coordinator, programs with no email address listed, and fellowship programs not participating in the 2020–2021 match cycle. During the 2020–2021 Match cycle, there were a total of 265 ACGME Accredited Fellowship Programs and 285 ACGME Accredited Obstetrical and Gynecological Residency Programs. Of the total number of programs, 115/265 (43.4%) Fellowship Programs and 122/285 (42.8%) met inclusion criteria and were sent surveys.

Surveys were distributed to each party after the conclusion of each specialty’s respective interview season. Each recipient invited to participate in this research study was incentivized by a $5.00 virtual gift card on completion of the survey. Participants had an option of remaining anonymous and were provided with explicit information stating that their responses were being utilized for research purposes. Respondents were de-identified upon completion and data was stored in a secure database

The investigators (JMP, MK, and NAL) were involved in the construction of the three survey tools. JMP has been an Obstetrics and Gynecology Residency Program Director for eleven years and has extensive experience in resident recruitment and the interview process; MK was a fellowship applicant and had personal experience with the virtual interviews, and NAL is a Clinical Research Fellowship Program Director with six years of experience in both resident and fellowship recruitment. The investigators developed survey tools based on existing literature, knowledge of current residency and fellowship interview practices, discussions with experts in the field, and their own experience in resident recruitment. In addition, the authors utilized data from the National Residency Match Program (NRMP) Specialties Matching Service Program Director Survey which contains information on what factors program directors consider important in ranking applicants [15]. All three investigators reviewed the survey tools to provide feedback on the relevance and clarity of questions, and projected time needed to complete the survey.

The Fellowship and Residency Program directors survey tools underwent a content validation process with faculty members that regularly participate in resident and fellowship recruitment. Based on these results, which showed an appropriate range of variance, we further refined the survey, including incorporating questions on the convenience of the virtual format. The survey tool was then piloted by a group of 5 program directors of varying specialties that closely resemble the targeted recipients. The results of the pilot resulted in no substantive changes in survey content, but several questions were edited for clarity based on this feedback.

The final survey tools contained 11–13 four-point Likert scale questions evaluating demographic factors as well as several factors regarding the interview and matching process, and up to four free response questions (Appendix 1). Likert questions used a four-point Likert scale (i.e., where 1 = strongly disagree and 4 = strongly agree) as an ordinal variable. A four-point Likert scale was deliberately chosen to avoid neutral responses to questions.

Data were analyzed using the Statistical Package for the Social Sciences (SPSS; IBM version 27). Likert data were analyzed by computing means, and response distributions, for each question to compare self-reported participant agreement or disagreement with survey statements. For qualitative analysis of free response questions, JMP, MK, and NAL reviewed all qualitative data individually to identify salient themes. They pooled their initial analyses and reviewed the qualitative together until no new themes emerged. The final illustrative quotes were chosen to represent all salient themes by a majority consensus of investigators.

## Results

### Fellowship program directors

A total of 265 ACGME accredited Ob/Gyn fellowship programs participated in the 2020–2021 Match cycle. A total of 115 questionnaires were sent to REI, MFM, Gyn/Onc, and FPMRS fellowship program directors meeting inclusion criteria (115/265, 43.4%). Overall, 2 (1.7%) opted-out, 4 (3.4%) ‘bounced back’ and 75 of the 111 (67.6%) fellowship program directors that received the survey completed it. Fellowship PDs that completed our survey included: MFM (n = 37/75, 49.3%), Gyn/Onc (n = 14/75, 18.6%) FPMRS (n = 13/75, 17.3%), and REI (n = 11/75, 14.6%). Based upon the total number of national Ob/Gyn fellowship programs (n = 265), MFM had the greatest representation in this survey n = 37/97, 38.1%), followed by FMPRS (n = 13/54, 24.1%), GynOnc (n = 14/63, 22.2%) and REI (n = 11/51, 21.6%). Each respondent had varying amounts of experience in the position of Fellowship Program Director, ranging a minimum of 1 year to a maximum of 30 years (mean 10.1 years). Nearly half (n = 37, 49.3%) of the fellowship program directors had experience using a virtual platform for education or business prior to the start of the Covid-19 pandemic. A virtual social event or virtual meet-and-greet was held by 48% (n = 36) of fellowship programs.

**Figure 1.**
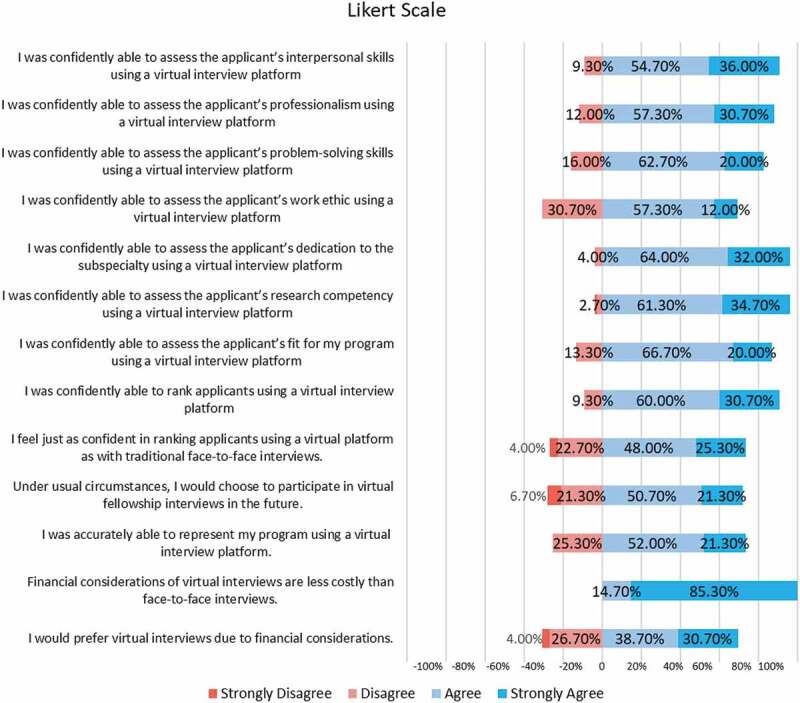
Likert survey for fellowship program directors.

Of the fellowship program directors surveyed, 90.7% (n = 68) strongly agreed or agreed that they could confidently assess the applicants’ interpersonal skills using a virtual platform, and 88% (n = 68) could confidently assess an applicant’s professionalism (Figure 1). However, 77.3% (n = 58) program directors felt that they were unable to accurately represent their own program using a virtual platform. Interestingly, 90.7% (n = 68) of fellowship program directors agreed or strongly agreed that they felt confident in making a rank list after their virtual interview season. However, when asked if they were just as confident in preparing a rank list after virtual interviews as they have been with traditional interviews in the past, only 73.3% (n = 55) agreed while 22.7% (n = 17) disagreed and 3% (n = 3) strongly disagreed. Going forward, 72% (n = 54) strongly agreed or agreed that in the future, even under normal circumstances, they would choose to participate in virtual interviews. Finally, one thing that everyone agreed on was that virtual interviews cost less, with 100% (n = 75) of respondents agreeing or strongly agreeing with this statement. Figure 1 presents responses to all thirteen Likert survey questions. Appendix 2 presents results of the Likert survey, stratified by type of subspeciality fellowship program.

The fellowship program director responses to the free text questions revealed key themes for the first two free text questions. When asked ‘What did you like best about the virtual interview process?’ themes identified were: Cost-savings for applicants, Ability to reach more applicants, and Convenience. When asked ‘What was most challenging about the virtual interview process?’ themes identified included: Inability to assess fit for the program, Not being able to showcase facility, Inability to gauge applicants’ interest, and Adjusting to a new format. Key themes and selected quotes are shown in Table 1.

**Table 1.** Fellowship program director free text response questions themes and selected quotes

Identified Theme	Selected quotes
What did you like best about the virtual interview process?	
Efficiency	‘It ran on time–because the breakout rooms made you finish on time’ ‘Quick, group format, easier. Achieved the same goals with less headache.’ ‘Ease of flowing from one interviewee to the next, without waiting for them to move from room to room’
Cost-savings for applicants	‘I prefer in-person, but I appreciate the financial benefit to applicants’ ‘The applicants saving of money and time’ ‘The applicant’s financial benefits: For that reason alone, I would support virtual interviews despite possible limitations in assessment of the candidates.’
Reaching more applicants	‘The ability to interview candidates from other states that would probably not have interviewed due to cost.’ ‘Greater acceptance of interview invitations.’ ‘We expanded our applicant pool because, with in person interviews, applicants on the west coast would have to take 3 days off to come interview. With virtual interviews, we can interview any candidate.’ ‘It allowed all applicants an even playing field for interviewing. They were not restrained by financial or COVID quarantines. Also due to lack of traveling may have accepted more interviews because they were able to attend.’
Convenience	‘It seemed easier to make arrangements. All the applicants looked rested.’ ‘It was easy for applicants. I didn’t find it a barrier to assess the applicants.’ ‘Faculty able to interview at any location/site during their day.’ ‘Less disruptive. Did not have interviews on Saturday, which faculty loved.’ ‘More convenient for applicants. Faculty who wanted to participate in the interview despite being on vacation were able to participate remotely. Ability to interact with candidates was better than expected.’
What was most challenging about the virtual interview process?	
Inability to assess interpersonal skills	‘Feeling confident that we can assess candidates remotely as well as in person’ ‘The “down time” more casual chatting was eliminated – I’m not sure I had quite as good of a feel for personality.’ ‘Inability to see applicants during the less formal parts of interviews (socializing, interactions with staff and with each other). That is certainly missing from virtual and is important in assessing a person.’ ‘Unable to assess body language and interaction with other people in a group’ ‘Interaction with other applicants, non-physicians, body language are also import clues to character especially when looking for good fit’ ‘Less opportunity to interact informally at dinner and no one-on-one interaction with current fellows in between interviews to get fellow feedback on communication and personality’ ‘Not having the get together the night before prevented me from seeing their natural interaction in a group.’ ‘IT (Informational Technology) is fatiguing. I like a group social interaction, that was not as easy virtually. The candidates did not get the feel of the local area, the lab facilities, the restaurant scene without a visit’ ‘Getting a sense for how they interact in a group and with staff’ ‘Fellows usually have lunch with candidates and have a chance to meet them and speak informally. We tried to do it virtually, but it doesn’t work as well for casual conversation’
Not able to showcase facility	‘Convincing them that they would like to live in a city/state they have never seen’ ‘Not able to showcase our campus and facilities as well as we would have done with in-person interviews.’ ‘Cannot get a feeling of the person and the person cannot get a feeling of the city or program’ ‘Trying to convey the spirit of the program to applicants.’ ‘I am not sure they were able to experience our true culture and our program’
Inability to gauge the applicant’s interest	‘Unable to gauge how serious they were about us’ ‘Ability of any candidate to apply to every program without worrying about travel/cost/time’
Adjusting to new format	‘Learning the best way to do the interviews, etc. But once learned, it went great and can’t imagine going back.’ ‘Sitting in front of a computer screen for prolonged periods is a challenge.’ ‘It was somewhat awkward at times. It was more exhausting to interview via Zoom for half a day than in person. Although we had a happy hour, it was not the same as happy hours we have in person.’ ‘Zoom Fatigue!’

When asked ‘Were virtual interviews more convenient or less convenient to your fellowship program’, 72.0% (n = 52) of fellowship program directors responded that virtual interviews were more convenient for their program, 21.3% (n = 16) felt that they were no different than in-person interviews, and 6.7% (n = 5) believed that they were less convenient. Of the 52 program directors that found virtual interviews to be more convenient for their program, five further commented that the increased convenience of virtual interviews came with trade-offs, including being ‘less informative’, ‘less effective’, ‘less valuable’, ‘less useful’, as well as ‘less satisfying and more anxiety-provoking’. For those finding virtual interviews less convenient for their program, free text comments revealed that this sentiment was attributed to ‘adjusting to a new format’ and ‘additional [interview] days and more interviews’. One further elaborated on the factors associated with the novelty, stating, ‘It was less convenient, but I think that was because it was novel, and there were a lot of logistics … .for example, practicing with faculty, determining the questions, ensuring schedules, and making a program video that won’t be as necessary in coming years if we continue with a virtual interview platform.’ Regarding decreased convenience and novelty, another also commented, ‘Slightly less convenient because it was new, but less expensive and ultimately more efficient.’

### Technical difficulties

With respect to fellowship programs, most had a positive experience with using a virtual platform. A total of 25.3% (n = 19) programs reported experiencing a technical difficulty, but most were minor. Twelve programs experienced minor connectivity problems with individual applicant(s). In all cases, a follow-up telephone call allowed the interview to continue. A few programs experienced initial difficulty transitioning applicants between breakout Zoom rooms, but this was resolved with increasing experience on the platform. Three programs experienced a major problem, including, 1) a major storm that temporarily knocked out power on the day of the interview, 2) hospital Wi-Fi was not working on the first day of interview, and 3) obtaining an inadequate Zoom License contract that supported only 5 Zoom connections per user. However, these programs were able to overcome these challenges using ‘every other technology available to us, including but not limited to hotspots, FaceTime, [and] just good old telephone conversation!’

### Residency program directors

A total of 285 ACGME accredited residency programs participated in the 2020–2021 Match cycle, of which 122/285 (42.8%) met inclusion criteria and were sent a survey. Of 122 surveys that were sent to Ob/Gyn residency program directors across the nation, 5 (4.1%) bounced-back, 1 (0.008%) opted-out, and a total of 67/117 received surveys (57.3%) were completed. Mean years of experience as a residency program director was 4.8 years (range 1–15 years). In the 2020–2021 interview cycle, 77.6% of program directors reported having residents applying for Ob/Gyn fellowships. The mean number of residents applying from these programs was 1.93 (range 1–9). When asked if they typically limit the number of in-person interviews a resident can accept, 94% (n = 63) did not establish a limit, while the remaining program directors set a limit that ranged from 6–28 interviews.

**Figure 2.**
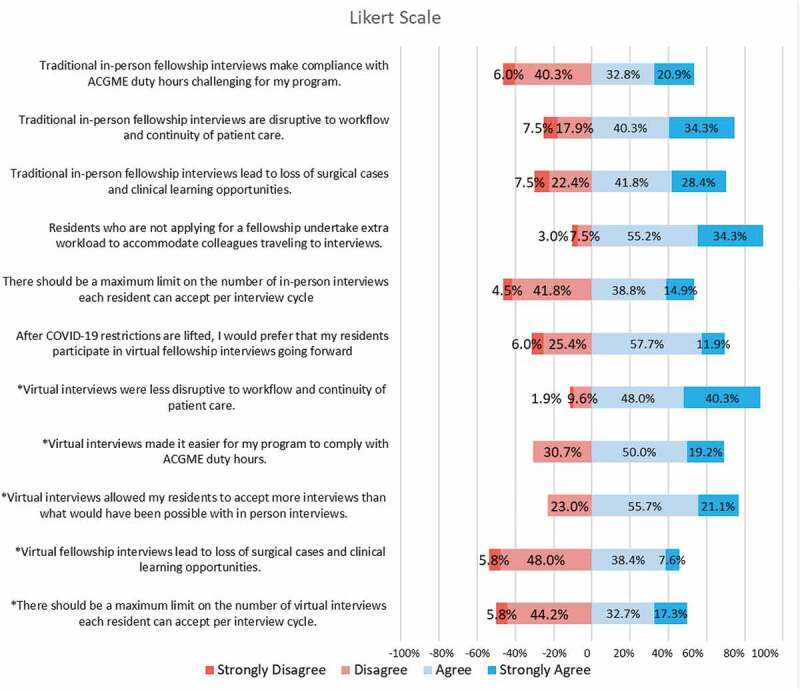
Likert survey for residency program directors (*Answered only by program directors who had residents applying for a fellowship during the 2020–2021 virtual match. (N = 52).

All program directors were asked questions concerning the impact that traditional in-person fellowship interviews have on their residency programs. 53.7% (n = 35) of the Residency Program Directors agreed or strongly agreed that traditional in-person fellowship interviews make compliance with Accreditation Council for Graduate Medical Education (ACGME) duty hours challenging for their program. Most (89.5%, n = 60) agreed or strongly agreed that with traditional in-person interviews, residents who are not interviewing undertake extra workload to accommodate the travel arrangements of those who are (Figure 2). Due to the travel requirements of in-person interviews, 74.6% (n = 50) of program directors reported that it disrupts workflow and continuity of patient care, and 70.2% (n = 47) believe that it causes residents to lose out on surgical and clinical learning opportunities. Finally, 53.7% (n = 35) believed that there should be a maximum limit on the number of in-person interviews an applicant can accept each interview cycle.

The 52 program directors who stated they had residents applying for the 2020–21 Match season were then asked further questions about the impact that virtual interviews had on their program. Most (88.5%. n = 46) agreed or strongly agreed that compared to in-person interviews, the virtual format was less disruptive to workflow and continuity of patient care. Over two-third (69.2%, n = 36) believed that virtual interviews made it easier for their program to comply with the ACGME duty hours, and 76.9% (n = 40) stated that it allowed their residents to accept more interviews than an in-person format. However, 46.2% (n = 24) of program directors still felt that virtual interviews led to loss of surgical cases and clinical learning opportunities and 50.0% (n = 26) believed that there should be a maximum limit on the number of virtual interviews an applicant can accept. Finally, when asked ‘After COVID-19 restrictions are lifted, I would prefer that my residents participate in virtual fellowship interviews going forward’ 60.2% (n-36) of program directors agreed or strongly agreed with the statement. Figure 2 depicts the Residency Program Directors responses to all eleven Likert survey questions.

## Discussion

Satisfaction

The Fellowship PDs responses to our survey revealed several findings with regard to their satisfaction with virtual interviews. Key themes identified from free-text responses were convenience, ability to reach more applicants, and cost-savings. With regards to convenience, many Fellowship PDs appreciated that a virtual platform, such as Zoom break-out rooms, kept the schedule on track because ‘the time slot for interviewing was fixed and the sessions just ended’. Additionally, Fellowship PDs liked the flexibility of scheduling, not needing to ‘run from room to room’, as well as the fact that faculty and fellows were able to interview from remote sites throughout the day. Many Fellowship PDs also commented that they appreciated the convenience afforded to applicants with respect to not needing to travel and ease of intervening from a familiar environment. Another important finding of our study was that the virtual format allowed Fellowship PDs to reach more geographically diverse applicants, and that they were more likely to accept interview invitations since they did not have to travel. This may be beneficial for programs that are less competitive or more geographically inaccessible. As one PD said, ‘More applicants were likely to interview at our institution than might have if they had to pay to fly here. I might not have been on the top of their list to consider.’

Additionally, based on free-text responses, many fellowship directors also recognized the financial benefit to applicants. Several studies have documented the financial burden associated with in-person interviews faced by fellowship applicants. A cohort of 269 applicants that participated in the Gynecologic Oncology fellowship match from 2008–2016, spent an average of $6,000 (range: $0–25,000), using personal savings (54%), credit cards (51%), family support (11%) or personal loans (3%) to cover costs of traditional in-person interviews [3]. A more recent study of 44 REI fellowship applicants found an average spending of $5,660 per applicant (range $900-15,000) [12]. With the Association of American Medical Colleges reporting in 2019 a median student debt of $200,000 after graduation, virtual interviews may reduce additive costs to pre-existing economic burden for fellowship applicants [13]. Fellowship programs also incur substantial costs during the interview season. A survey of General Surgery Program Directors representing over 600 programs reported mean hard costs of in-person recruitment, not including personnel effort, as approximately $8,400 per program [14]. Our study found that 100% of Fellowship PDs believed that their program saved money using a virtual platform. However, it is unclear if this would influence the use of a virtual interview format in the future.

Challenges

However, several challenges also emerged. Fellowship PDs found it difficult to assess applicants’ interpersonal skills, showcase their programs virtually, and determine if applicants were genuinely interested in their program. Although the Likert survey responses indicated that 90% of fellowship directors believed they could confidently assess an applicant’s interpersonal skills using a virtual platform (Figure 1), free-text responses indicated the virtual experience had shortcomings compared to face-to-face interactions (Table 1). Several of these challenges were associated with the inherent limitations of a completely virtual platform; as one Fellowship PD noted, ‘Zoom is not personal contact, which is helpful in the correct assessment of an applicant.’ Specific challenges included the limited ability to observe how applicants interact with staff, faculty, fellows, and each other. Additionally, the informal interactions that might occur in-between interviews, other down time, or during social events were non-existent. This may have limited the ability of some program directors to fully assess and utilize those factors which they have traditionally deemed most important to determine ‘fit’ [15].

The ease of scheduling virtual interviews also presented unique challenges to Fellowship Directors. Some expressed difficulty in assessing genuine interest in their program as applicants did not need to make a significant investment of time and/or money for travel to attend. The ability of a program director to gauge an applicant’s true interest when formulating their Rank Order List (ROL) is an important factor for several reasons. It is advantageous for PDs to rank highly those applicants who they deem are genuinely interested in their specific program because it increases the likelihood of a successful match. Overall, we found that almost 90% of Fellowship PDs claimed they could confidently prepare their rank order list at the conclusion of the virtual interviews. However, when asked if they were just as confident in preparing a rank list after virtual interviews as they had been with traditional interviews in the past, this number dropped to 73.3%. One Fellowship Director summarized their difficulty by saying: ‘Trying to gauge people’s interest … nothing says interest like spending some money to fly somewhere’. Another PD commented, ‘[I] would wager a program will be more apt to take a candidate from within the program … we definitely would have considered the insider first since the person would have been a known entity.’ Obviously gauging applicants’ interest is an important factor in the match process; the use of an allocation of signals to programs to indicate genuine interest in a program has been piloted and may prove of use in this regard [16].

Another key challenge encountered by Fellowship PDs was the inability to showcase their program virtually. This included not being able to adequately represent their hospital’s facilities virtually and also to convey the culture of their program. Additionally, PDs were not able to properly convey the social life of the town where the program is located. Given that virtual interviews will persist, more tools could be developed to convey program facilities, neighborhood, and intangible aspects of the program to applicants. This may include the use of virtual tours of the neighborhood showcasing popular spots such as gyms, parks, and restaurants, videos featuring ‘A Day in the Life of a Fellow’, as well as links to the program’s social media accounts.

Approximately half of the fellowship programs that responded to our survey hosted a virtual social event as part of the virtual interview schedule. These virtual ‘meet-and-greets’ were usually held in close proximity, either before or after the scheduled interview date. However, free text responses revealed that these virtual events did not replace typical social interactions. One program director stated, ‘We tried to do it [social event] virtually, but it doesn’t work as well for casual conversation’ and another declared, ‘Although we had a happy hour, it was not the same as happy hours we have in person’. (Table 1). Improving the structure of the social portion of the interview process to allow for more natural interaction between all participants should be a goal for future virtual interviews. In our experience, applicants use social programming with trainees alone to gauge their compatibility with potential future colleagues and to query trainees regarding sensitive topics such as vacation time, call schedules, faculty interactions, etc. Therefore, we feel that there is value to time spent amongst interviewees and trainees without program leadership present. This time affords applicants an opportunity to get a glimpse into the daily routine and more informal and social aspects of the program.

Residency Program Directors’ Take

The majority of the interviews for the obstetrics and gynecology subspecialty fellowships occur from April through September. Furthermore, many interviews occur in July, when the new academic year for residency programs begin. Shortages in the workforce at this time can prove especially challenging due to promotion of current residents and onboarding of new interns. In a study by the Society of Gynecologic Oncology of the 2008–2016 match cycles, applicants spent a mean of 15 (range: 0–45) days away from residency activities and 37% reported difficulty arranging coverage [3]. Similarly, a study of Orthopedic surgery applicants reported that residents missed an average of 11 days of clinical training for fellowship interviews and this was recognized to cause a high level of disruption by residency program directors [2]. We found that most residency program directors agreed or strongly agreed that traditional in-person fellowship interviews not only make compliance with ACGME duty hours challenging for their program, but also disrupts workflow and continuity of patient care. Specifically, the time away from the program may cause resident applicants to lose out on surgical and clinical learning opportunities. In addition, their resident colleagues, who may not be applying for a subspecialty fellowship, may have to undertake extra duty hours or on-calls to accommodate them, as noted by 89% of Residency PDs we surveyed. We found that over half the Residency PDs surveyed (53%, Table 1) agree that there should be limits on the number of in-person interviews a resident can accept, ranging from a 6–28 interview limit. Due to these practical considerations and residency logistics, fellowship applicants may not be able to accept all interview offers and fail to capitalize on important opportunities. For example, for REI applicants participating in the 2018 match, approximately 68% of residents reported missing an opportunity to interview at a program that they were interested in [12]. The most common reasons were two programs having the same interview date, applicants not able to travel due to geographic location, and cost was too high.

Virtual interviews may eliminate many of these barriers, However, this may have unintended consequences for some applicants, as one fellowship program director commented, ‘I think the top candidates get to accept more interviews virtually as compared to if they had to travel. This reduces the number of opportunities for moderate/marginal candidates.’ Additionally, we found that 46.2% of Residency PDs we surveyed still felt that virtual interviews led to loss of surgical cases and clinical learning opportunities, and 50% believed that there should be a maximum limit on the number of virtual interviews an applicant can accept. Capping the number of virtual interviews has been suggested for residency interviews, and is a subject of controversy [17]. Assessing whether virtual interviewing creates potential inequities in the match process, as well as further investigation into the consequences of capping the number of virtual interviews may be necessary in the future. It is important moving forward to consider other ways of ‘leveling the playing field” for applicants as well as programs. One possibility is changing the timing of interviews, e.g., shortening the interview season, and avoiding the month of July, when there is more stress in both the residency and fellowship programs. It is the PDs responsibility to ensure that their residents’ training is not compromised by excessive absence, but also to fully support the resident’s career aspirations. This is true whether interviews are virtual or in-person. We leave it to future researchers to delve into ways to mitigate these disturbances.

Going Forward

The majority of Fellowship PDs surveyed (72%) in our study responded that, despite some of the challenges attributed to the virtual format, they would elect to participate in a virtual interview season in the future. Similarly, from the perspective of Residency PDs, 60.2% favored their residents participating in virtual interviews in the future. However, one limitation of our survey tool was that we did not specifically solicit Fellowship PDs opinion on a ‘hybrid’ interview option. It is unclear how a ‘hybrid’ option would look. One fellowship PD suggested that ‘ I would be willing to do a two step interview, first virtual and select fewer numbers for a second [in-person] interview’. However, it is quite possible a hybrid option necessitating multiple rounds of interviews may increase workload, especially for some smaller, lower resource programs, and thus would not be favored by all. Other hybrid suggestions included allowing applicants a choice of attending an in-person or virtual interview. However, this option may have unintended consequences, potentially disadvantaging applicants who may not be able to travel in-person.

Strengths/Weaknesses

Strengths of our study include the use of two survey tools that assess the different, but complementary perspectives of Fellowship Program Directors and Residency Program Directors. We included a diverse population of Residency Program Directors from all over the country, as well as Fellowship Program Directors representing four of the major Ob/Gyn subspecialties. Additionally, our survey included qualitative data in the form of free-text responses to gather a more detailed and subjective view of their experiences. However, our survey methodology enabled only PDs that provided an email address to be surveyed. Thus, our response rate may be misleading when considered in relation to the total number of ACGME accredited programs. Additionally, our survey may be influenced by response bias in that PDs not providing email addresses may have different opinions that those that do, or PDs with especially strong thoughts on virtual interviews may be more likely to respond to the survey. Nevertheless, our response rate compares favorably with that of other national program surveys such as the NRMP Program Directors Survey 2021, which ranged from 13.5% to 32.7% [18].

## Conclusion

Changes forced by the Covid-19 pandemic have created an opportunity to re-think how to best recruit prospective Ob/Gyn fellowship candidates during forthcoming interview seasons. Benefits to applicants using a virtual platform included financial savings, convenience, and ability to accept more interviews. Residency Program Directors found it to be less disruptive to their program. A majority of Fellowship Program Directors were confident in preparing a rank order list after their virtual interview season. Lack of social interaction and difficulties in assessing fit may be the biggest challenges for future implementation. Finally, virtual interviews may have unintended consequences. Due to lack of financial and travel constraints, highly qualified applicants may accept more interview offers, thereby disadvantaging the remaining applicant pool. This could potentially lead to inequities in the match process. Further investigation will be required as we continue to use virtual interviews in the fellowship match process.

## Appendix 1

Liker Survey for Fellowship Program Directors. Percentages for REI, MFM, Gyn/Onc, FPRMS, corresponds to column % for within each specialty; Total represents combined the N from each specialty. REI: Reproductive Endocrinology and Infertility, MFM: Maternal Fetal Medicine, GYN ONC: Gynecologic Oncology, FPMRSL Female Pelvic Medicine and Reconstructive Surgery

**Table:**

	Strongly Agree					Agree					Disagree					Strongly Disagree				
	REI N (%)	MFM	GYN ONC	FPMRS	Total	REI	MFM	GYN ONC	FPMRS	Total	REI	MFM	GYN ONC	FPMRS	Total	REI	MFM	ONC	FPMRS	Total
I was confidently able to assess the applicant’s **interpersonal skills** using a virtual interview platform	2 (18.2)	10 (27.0)	4 (28.6)	11 (84.6)	27 (36.0)	7 (63.6)	24 (64.9)	8 (57.1)	2 (15.4%)	41 (54.7)	2 (18.2)	3 (8.1)	2 (14.3)	0	7 9.3	0	0	0	0	0
I was confidently able to assess the applicant’s **professionalism** using a virtual interview platform	1 (9.1)	10 (27.0)	3 (21.4)	9 (69.2)	23 (30.7)	6 (54.5)	25 (67.6)	8 (57.1)	4 (30.8)	43 (57.3)	4 (36.4)	2 (5.4)	3 (21.4)	0	9 (12.0)	0	0	0	0	0
I was confidently able to assess the applicant’s **problem-solving skills** using a virtual interview platform	1 (9.1)	8 (21.6)	0	6 (46.20	15 (20.0)	8 (72.7)	23 (62.2)	9 (64.3)	7 (53.8)	47 (62.7)	1 (9.1)	6 (16.2)	5 (35.7)	0	12 (16.0)	1 (9.1)	0	0	0	0
I was confidently able to assess the applicant’s **work ethic** using a virtual interview platform	1 (9.1%)	5 (13.5)	1 (7.1)	2 (15.4)	9 (12.0)	4 (36.4)	23 (62.2)	6 (42.9)	10 (76.9)	43 (57.3)	6 (54.5)	9 (24.3)	7 (50.0)	1 (7.7)	23 (30.7)	0	0	0	0	0
I was confidently able to assess the applicant’s **dedication to the subspecialty** using a virtual interview platform	2 (18.2)	9 (24.3)	3 (21.4)	10 (76.9)	24 (32.0)	8 (72.7)	26 (70.3)	11 (78.6)	3 (23.1)	48 (64.0)	1 (9.1)	2 (5.4)	0	0	3 (4.0)	0	0	0	0	0
I was confidently able to assess the applicant’s **research competency** using a virtual interview platform	2 (18.2)	12 (32.4)	2 (14.3)	10 (76.9)	26 (34.7)	7 (63.6)	24 (64.9)	12 (85.7)	3 (23.1)	46 (61.3)	1 (9.1)	1 (2.7)	0	0	2 (2.7)	0	0	0	0	1 (1.3)
I was confidently able to assess the applicant’s **fit for my program** using a virtual interview platform	1 (9.1)	7 (18.9)	1 (7.1)	6 (46.2)	15 (20.0)	6 (54.5	28 (75.7)	9 (64.3)	7 (53.8)	50 (66.7)	4 (36.4)	2 (5.4)	4 (28.6)	0	10 (13.3)	0	0	0	0	0
I was confidently able to **rank** applicants using a virtual interview platform	1 (9.1)	12 (32.4)	2 (14.3)	8 (61.5)	23 (30.7)	7 (63.6)	23 (62.2)	10 (71.4)	5 (38.5)	45 (60.0)	3 (27.3)	2 (5.4)	2 (14.3)	0	7 (9.3)	0	0	0	0	0
I feel **just as confident in ranking of applicants using a virtual platform** as with traditional face-to-face interviews.	0	10 (27.0)	1 (7.1)	8 (61.5)	19 (25.3)	4 (36.4)	22 59.5	5 (35.7)	5 (38.5)	36 (48.0)	4 (36.4)	5 (13.5)	8 (57.1)	0	17 (22.7)	3 (27.3)	0	0	0	3 (4.0)
Under usual circumstances, **I would choose to participate in in virtual fellowship** interviews in the future.	1 (9.1)	9 (24.3)	3 (21.4)	3 (23.1)	16 (21.3)	4 (36.4)	21 (56.8)	6 (42.9)	7 (53.8)	38 (50.7)	3 (27.3)	7 (18.9)	3 (21.4)	3 (23.1)	16 (21.3)	3 (27.3)	0	2 (14.3)	0	5 (6.7)
I was accurately able to **represent my program** using a virtual interview platform.	1 (9.1)	7 (19.4)	3 (21.4)	5 (38.5)	16 (21.3)	4 (36.4)	22 (61.1)	6 (42.9)	7 (53.8)	39 (52.0)	6 (54.5)	7 (19.4)	5 (35.7)	1 (7.7)	19 (25.3)	0	0	0	0	0
Financial considerations of virtual interviews are **less costly** than face-to-face interviews.	10 (90.9)	30 (81.1)	11 (78.6)	13 (100.0)	64(85.3)	1 (91.)	7 (18.9)	3 (21.4)	0	11 (14.7)	0	0	0	0	0	0	0	0	0	0
I would **prefer virtual interviews** due to **financial considerations.**	2 (18.2)	12 (32.4)	4 (28.6)	5 (38.5)	23 (30.7)	3 (27.3)	15 (40.5)	5 (35.7)	6 (46.2)	29 (38.7)	3 (27.3)	10 (27.0)	5 (35.7)	2 (15.4)	20 (26.7)	3 (27.3)	0	0	0	3 (4.0)

## Appendix 2

For the Fellowship Program Director Questionnaire, participants were asked how much they agreed or disagreed with the following 13 statements using a 4-point Likert scale:

1. I was confidently able to assess the applicant’s interpersonal skills using a virtual interview platform
2. I was confidently able to assess the applicant’s professionalism using a virtual interview platform
3. I was confidently able to assess the applicant’s problem-solving skills using a virtual interview platform
4. I was confidently able to assess the applicant’s work ethic using a virtual interview platform
5. I was confidently able to assess the applicant’s dedication to the subspecialty using a virtual interview platform
6. I was confidently able to assess the applicant’s research competency using a virtual interview platform
7. I was confidently able to assess the applicant’s fit for my program using a virtual interview platform
8. I was confidently able to rank applicants using a virtual interview platform
9. I feel just as confident in ranking of applicants using a virtual platform as with traditional face-to-face interviews.
10. Under usual circumstances, I would choose to participate in virtual fellowship interviews in the future.
11. I was accurately able to represent my program using a virtual interview platform
12. Financial considerations of virtual interviews are **less costly** than face-to-face interviews
13. I would prefer virtual interviews due to financial considerations.

Free response questions included the following:

1. What did you like best about the virtual interview process?

2. What was most challenging about the virtual interview process?

3. Were virtual interviews more convenient or less convenient to your fellowship program?

4. Did you experience technical difficulties? If so, please elaborate on the number and nature of these technical difficulties.

For the Ob/Gyn Residency Program Director questionnaire, participants were asked how much they agreed or disagreed with the following 11 statements using a 4-point Likert scale:

1. Traditional in-person fellowship interviews make compliance with ACGME duty hours challenging for my program.
2. Traditional in-person fellowship interviews are disruptive to workflow and continuity of patient care.
3. Traditional in-person fellowship interviews lead to loss of surgical cases and clinical learning opportunities.
4. Residents who are not applying for a fellowship undertake extra workload to accommodate colleagues traveling to interviews
5. There should be a maximum limit on the number of in-person interviews each resident can accept per interview cycle
6. After COVID-19 restrictions are lifted, I would prefer that my residents participate in virtual fellowship interviews going forward
7. Virtual interviews were less disruptive to workflow and continuity of patient care.
8. Virtual interviews made it easier for my program to comply with ACGME duty hours.
9. Virtual interviews allowed my residents to accept more interviews than what would have been possible with traditional face-to-face interviews
10. Virtual fellowship interviews lead to loss of surgical cases and clinical learning opportunities.
11. There should be a maximum limit on the number of virtual interviews each resident can accept per interview cycle.
